# Associations between Body Mass Index and Urban “Green” Streetscape in Cleveland, Ohio, USA

**DOI:** 10.3390/ijerph15102186

**Published:** 2018-10-06

**Authors:** Xiaojiang Li, Debarchana Ghosh

**Affiliations:** 1 Senseable City Lab, Department of Urban Studies and Planning, Massachusetts Institute of Technology, 9-250, 77 Massachusetts Avenue, Cambridge, MA 02139, USA; 2 Department of Geography, University of Connecticut, Storrs, CT 06269, USA; debarchana.ghosh@uconn.edu

**Keywords:** Body mass index (BMI), walkability, street greenery, Google Street View

## Abstract

Public health researchers are increasingly interested in assessing the impact of neighborhood environment on physical activities and chronic health issues among humans. Walkable streets and proximity to green space have long been believed to promote active lifestyles in cities, which contribute to positive health outcomes among residents. Traditionally, urban environmental metrics were calculated at the area level to describe the physical environment of neighborhoods. However, considering the fact that streets are the basic unit for human activities in cities, it is important to understand how the streetscape environment can influence human health conditions. In this study, we investigated the influence of street greenery and walkability on body mass index in Cleveland, Ohio, USA. Different from the area level and overhead view greenery metrics, we used the green view index calculated from the Google Street View to represent the amount of street greenery. The Walk Score was used to indicate the walkability of neighborhoods also at the street level. Statistical analysis results show that the Walk Score has a more significant association with decreased BMI for males than females and the street greenery has a more significant association with decreased BMI for females than males in Cleveland, Ohio. The results of this study would provide a reference for designing gender-specific healthy cities.

## 1. Introduction

Obesity rates have risen significantly in the last half-century in many countries [1]. It was estimated that in 2014, approximately 1.9 billion adults were considered as obese [2]. In the United States, the prevalence of obesity was 36.5% among adults during 2011–2014 [3]. Obesity causes many comorbidities, such as diabetes, hypertension and cardiovascular illness [2,4,5,6,7], and accumulates huge medical costs every year [3,8]. 

Physical inactivity is one of the major causes of obesity [9,10,11]. The social–ecologic theory of human behavior suggests that some environmental factors in cities influence the likelihood of being physically active [1,12,13], which would further influence obesity. These environmental factors include both natural and built environment factors [1,12,14,15,16,17]. In a recent article, Sander et al. [1] found varying age–gender associations between body mass index (BMI) and urban green space in Ohio, U.S. The association between the BMI and the accessibility of urban green space was stronger for women and younger people. Hillsdon et al. [16] found that there is no significant association between physical activity and the accessibility of urban green spaces for middle-aged adults in Norwich, England where as Toftager et al. [17] reported a significant association between the distance to urban green spaces and both physical activity and obesity in Denmark. Coutts et al. [14] also found that living closer to urban parks would contribute a higher physical activity level at the county level in Florida, U.S. 

The built environment factors, such as higher housing density, existence of sidewalks, higher intersection density, easier access to transit, and greater land use mix, have been found to increase the walkability level and the frequencies and length of physical activities [13,18,19]. Specifically, Chiu et al. [12] found that people living in low-walkability areas with less opportunities to be active have a higher prevalence of overweight and obesity in Ontario, Canada. In contrast, Rundle et al. [20] found that residential neighborhoods with higher walkability usually have higher levels of active commute and physical activities such as walking and running. Casagrande et al. [21] studied the association of walkability with obesity directly in Baltimore city, Maryland. Results show that in predominately white and high-socioeconomic neighborhoods, those people residing in highly walkable neighborhoods tend to have a lower prevalence of obesity compared with people in less walkable neighborhoods. However, the association between walkability and obesity is not significant in less-affluent neighborhoods. 

Better understanding of the association of obesity with urban environment would provide an important reference for urban planning to reduce obesity rates [1]. Studies have investigated associations between obesity and accessibility to green spaces and urban built environment separately, thereby missing their combined effect. Very few studies investigated the association between urban and built environment together with obesity. In addition, several studies focused on the proximity to area level indicators such as urban green spaces or parks; but fewer studies investigated the street-level greenness of neighborhoods. The street-level greenness is more realistic and has a more direct connection with people while walking, running, or biking along the streets. 

In this study, we investigated the association between BMI of residents and the neighborhood greenness in Cleveland, Ohio for different age–gender groups. Both the natural and the built environment features were considered in the analyses. The green view index (GVI), calculated based on the Google Street View images captured at different horizontal view angles, indicates the greenness of the neighborhood. Different from previous area level green metrics, the GVI quantitatively represents how much greenery a pedestrian can see from a ground level, which may have more direct connection with human behaviors [22,23]. The Walk Score is added in the analyses as the indicator of the built environment because Walk Score generally indicates opportunities and potential for walking on a given street segment. It is calculated based on the proximity to various urban amenities and the density of such amenities [24,25]. 

## 2. Study Area and Dataset

**Figure 1:**
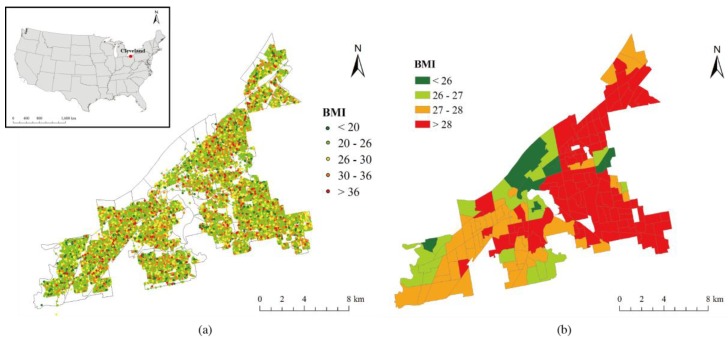
The spatial distribution of BMI samples at the site level and census tract level in Cleveland, Ohio, USA.

Cleveland is the second largest city in Ohio (Figure 1) and it has a population of 388,000. The BMI is used to measure the weight status in the study area. It is a summary measure of height and weight, and widely used for monitoring prevalence of overweight and obesity at individual and population levels. Based on the algorithm from the US Center for Disease Control Prevention, the BMI for residents was estimated by dividing an individual’s weight (kg) by the square of their height (m). The weights and heights were self-reported data from 305,295 residents (149,797 males, 155,498 females). The data was obtained from the Ohio Bureau of Motor Vehicles (BMV) driver’s license and state identification card applications with other useful variables such as age, gender, and residential location of residents. Considering the fact that around 87.2% of Ohio’s driving-age population possesses driver’s licenses, the dataset used in this study would be seen as generally representative of the overall population in the study area. 

Figure 1 shows the spatial distribution of BMI values in the study area at the sample sites and the census tract levels. In order to represent the neighborhood environment, we created sample sites every 50 m along the streets. These sample sites were further used to download Google Street View images and collect Walk Score. Based on the age and gender variables in the dataset, we categorized the census tract-level BMI for females and males for the following age groups: 18–29 (young adult), 30–50 (middle adult), 51–65 (older adult), and 65–84 (retiree).

## 3. Methodology

### 3.1. Green View Index Calculation Based on Google Street View

**Figure 2:**
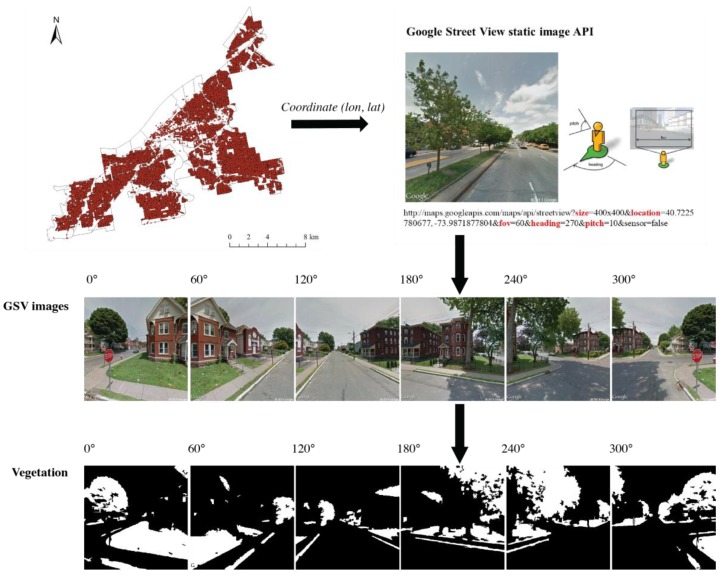
Google Street View (GSV) image collection and classification.

In this study, we used a green view index (GVI) to measure the greenness of neighborhoods. The GVI quantifies the visibility of the street greenery based on the street-level images [22]. To do this, for each sample site, we calculated the average percentage of greenery from six Google Street View (GSV) images at different directions (Figure 2). Based on the coordinates of those sample sites, we collected six static GSV images at six different horizontal directions at view angles of 0°, 60°, 120°, 180°, 240° and 300° for each sample site. Only those GSV images taken in leaf-on seasons were used in this analysis. We used the object-based image classification algorithm to extract the greenery from the static images [23]. Based on the classified GSV images, we further calculated the GVI for each site. Figure 2 shows the workflow for collecting GSV images and classifying vegetation from GSV images. 

Based on the image classification result, we further calculated the GVI using Formula (1),

(1) $$GVI=\frac{\sum_{i=1}^{6}Area_{g\_i}}{\sum_{i=1}^{6}Area_{t\_i}},$$

where *Area_g_i_* is the number of green pixels in a static GSV image, and *Area_t_i_* is the number of total pixels in one GSV image. The GVI indicates the average percentage of greenery pixels in six GSV images at six different horizontal directions that cover the 360° views. 

### 3.2. The Collection of Walk Score Data

We used the Walk Score to represent the walkability in the study area [24,25]. Walk Score is a metric to measure the walkability of neighborhoods based on the proximity to urban facilities and the density of various urban facilities. These urban facilities include built environment factors such as transitions, bus stops, grocery stores, parks, banks and other facilities to support more walking and biking than driving [24,25]. The Walk Score provides a convenient and inexpensive option in exploring the relationships between urban built environment, physical activity, and obesity [24]. The Walk Score data for all sample sites along the streets in the study area were collected through the Walk Score API by using the coordinates of those sites as the input.

### 3.3. Statistical Analysis 

In order to investigate the influence of street greenery (GVI) and walkability (Walk Score) on BMI, we conducted statistical regression models. Socioeconomic variables were included in the models as confounding factors. Based on previous studies [1,14,26], we selected per-capita income, percentage of Hispanics, percentage of African Americans, percentage of non-Hispanic whites, percentage of Asians, percentage of people with a Bachelor’s or higher degree, and percentage of people without a high-school diploma to indicate the socioeconomic status of the residents. All of these socioeconomic variables were derived from the American Census Survey 5-year estimates (2009–2014) at the census tract level.

In order to make the GVI, the Walk Score and the BMI data directly comparable to socioeconomic variables, we aggregated the site-level GVI map, Walk Score map and the BMI map to the census tract level by the mean value. To combine the effects of street greenery and walkability, an interaction term of GVI and Walk Score was also added in the statistical models. We first ran ordinary least-squares (OLS) regression models to analyze the associations between BMI and the independent variables (GVI, Walk Score, GVI*Walk Score, and confounding variables) for different age–gender groups. Next, we checked for spatial autocorrelation of residuals in order to control the effect of the spatial autocorrelation, if any. Results show that there is no significant spatial autocorrelation in the residuals. Therefore, spatial regression model was not conducted. 

## 4. Results

**Figure 3:**
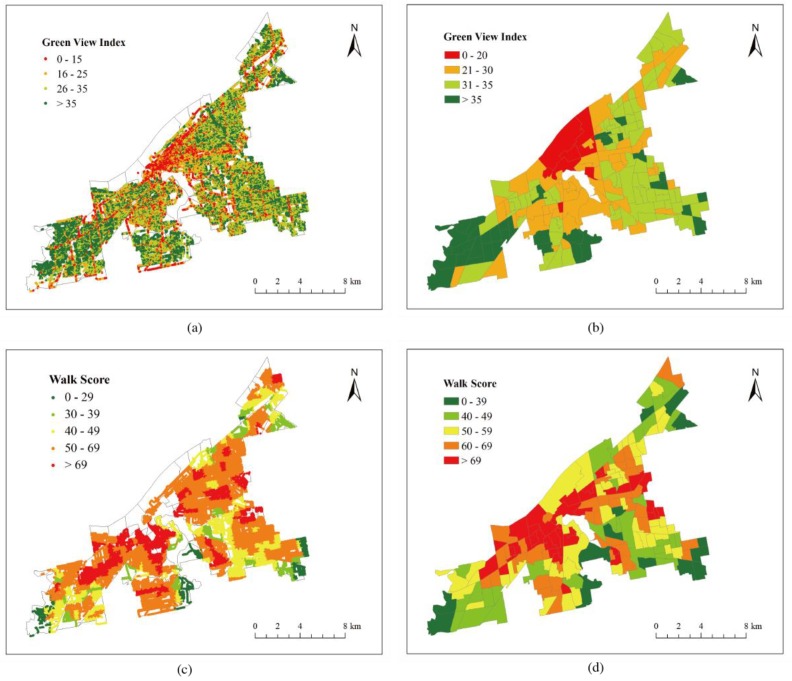
The spatial distributions of GVI and Walk Score in the study area, (**a**) spatial distribution of GVI at site level, (**b**) spatial distribution of GVI at census tract level, (**c**) spatial distribution of Walk Score at site level, (**d**) spatial distribution of Walk Score at census tract level.

Figure 3 shows the spatial distributions of the GVI and Walk Score at the site level and the census tract level. There is a clear spatial pattern of GVI with the central parts of the city showing lower GVI values compared with the peripheral areas (Figure 3a,b). In comparison to the spatial distribution of GVI, the Walk Score has a very different distribution (Figure 3c,d). The central parts of the city has higher Walk Score values than the peripheral regions of the city. 

Table 1 shows the correlation analysis results between the BMI and the independent variables. The Walk Score has a significant and negative correlation with BMI where as there is no significant correlation between GVI and BMI. Similar with many previous studies [26,27,28], the BMI has high and statistically significant correlations with the socioeconomic variables. African American residents have a very positive and significant correlation with the average BMI. However, the percentage of non-Hispanic whites, percentage of Asians and percentage of Hispanics all have significant and negative correlations with BMI. In addition, the BMI also has significant correlations with income and educational levels. The per-capita income and percentage of people with a Bachelor’s or higher degree are significantly and negatively correlated with BMI. The percentage of people without a high-school diploma has a positive and significant correlation with the BMI.

OLS regression model results show that the associations between the BMI and the independent variables vary among different age–gender groups. For the young females (18–29 years), the BMI has no significant association with both the Walk Score and the GVI (Table 2). Similar to the young female group, both the Walk Score and the GVI have no significant association with BMI for young males. The interaction term has no significant association with the BMI for both groups. 

Table 3 presents the OLS regression model results for females and males of middle-aged groups (30–50 years). For middle-aged females, the BMI has a significant and negative association with the GVI. There is no significant association between the BMI and the Walk Score. For middle-aged males, significant associations of BMI with both Walk Score and GVI were detected. The Walk Score is significantly and negatively associated with the BMI, but the GVI is significantly and positively associated with the BMI. For both groups, the interaction term is not significantly associated with the BMI.

The OLS regression analyses results for old-aged people (51–65 years) are presented in Table 4. For females, both the GVI and the Walk Score have no significant association with the BMI. For males, the GVI has no significant association with the BMI. The Walk Score has a weakly and significantly negative association with the BMI. The interaction term has no significant association with the BMI for both groups.

Table 5 gives the regression analysis results for retirees. For female retirees (66–84 years), the GVI has a significant and negative association with the BMI. There is no significant association between the Walk Score and the BMI. However, for male retirees, there is no significant association between the GVI and the BMI. The Walk Score also has no significant association with the BMI. For both groups, the interaction term is not significantly associated with the BMI.

## 5. Discussion 

Increasing walkability along streets and neighborhoods helps to promote an active lifestyle, which has long been recognized as beneficial to human health [12,20,25,29]. Analyzing the connection between urban natural and built environments and the obesity level of residents would be a perfect case for analyzing the interplay between obesity and urban environment. Previous studies conducted in different countries and regions show inconsistent associations between the neighborhood environment and obesity [16,29,30,31,32]. Rather than focusing on natural environment alone or one specific age–gender group of people, this study investigated the association between the urban natural environment and built environment on obesity measured by BMI for different age–gender groups. The street-level greenery and Walk Score were used to indicate the natural environment and built environment respectively at the neighborhood level. Different from previous studies that focus on large patches (area-level) of urban green spaces, this study used the GVI derived from the Google Street View to measure the greenness at the neighborhood level. The GVI is calculated based on street-level images and it is more suitable to represent the human exposure and daily experience of urban greenery [22]. Socioeconomic variables that were derived from census the data were used as the confounding variables to control for the effects of different social contexts on residents’ BMI. Results show that the greenness and walkability at the neighborhood level have different effects on different age–gender groups of people. Generally, the neighborhood greenness is significantly associated with decreased BMI for females, and there is no significant association between the BMI and neighborhood greenness for males. Different from the association of neighborhood greenness with BMI, the Walk Score is significantly associated with decreased BMI for males but not for females. For different age-groups of people, the GVI and Walk Score also have different associations with the BMI. For young people, regression results show that both Walk Score and GVI have no significant association with the BMI. For the middle-aged and retiree groups, the GVI is significantly associated with decreased BMI for females. However, the GVI is significantly associated with increased BMI for middle-aged males. For middle-aged and old-aged males, the Walk Score is associated with decreased BMI. The different associations of the urban natural and built environments with BMI among different age–gender groups may provide some references for creating more healthy neighborhoods. Increasing the greenery and walkability of neighborhoods would help to decrease the obesity for some age–gender groups. In addition, future urban planning should also consider the different effects of the urban environment on human lifestyles for different age–gender groups. 

Parks and other forms of green space are among the key environmental supports for recreational physical activity [14]. Guaranteeing the proximity to urban green space has also been considered as an important principle in urban planning. Sander et al. [1] analysis showed that in Cleveland, the association between the BMI and accessibility of green space is stronger for women and younger people. However, in this study, we found that the greenness of the neighborhood has a stronger association with the middle-aged and retiree groups. This could be explained by the fact that different green metrics measure urban greenery from different perspectives, and the GVI measures the neighborhood level greenness rather than the proximity to large patches of green space. It would be interesting to investigate the different functions of different types of urban green space in influencing human health conditions in future studies. 

There are limitations of this study that need to be discussed. Firstly, the current study was conducted at the census tract level in order to have more stable census data. However, using smaller geographical units would be better to reflect the connection between natural and built environments on individual BMI. This is because the physical environment would influence those people within walking distance, which means that finer geographic units or street-level analyses would better represent the interaction between human beings and the environment. However, getting reliable socioeconomic variables at the fine geographic level would be a challenge. The Walk Score could not fully represent the urban built environment and the walkability of neighborhoods. In future studies, more metrics describing the urban built environment should be considered for such analyses. 

## 6. Conclusions

This study found that associations between body mass index and Walk Score and Green View Index vary among different age–gender groups of people. The Walk Score has a more significant association with decreased BMI for males over females. The Green View Index has more significant association with decreased BMI for females than males, especially for middle-aged and retiree groups. The results point to an interesting conclusion that the existence of the urban greenery has a stronger correlation with BMI for females rather than males. 

## Figures and Tables

**Table 1:** The correlation analysis between the BMI and independent variables.

Independent variables	Coefficients	Sig. (2-Tailed)	*N*
Walk Score	−0.263 **	0.000	175
Green view index	0.056	0.463	
Percentage of African Americans	0.718 **	0.000	
Percentage of non-Hispanic Whites	−0.746 **	0.000	
Percentage of Asians	−0.504 **	0.000	
Percentage of Hispanics	−0.166	0.028	
Per Capita income	−0.718 **	0.000	
Percentage of people with a Bachelor‘s or higher degree	−0.800 **	0.000	
Percentage people without a high-school diploma	0.432 **	0.000	

** Correlation is significant at the 0.01 level (2-tailed).

**Table 2:** The statistical regression analysis results for females and males of the young-aged group (age 18–29).

Independent Variables	Coefficients		*N*
	Female (*t*-Value)	Male (*t*-Value)	
Walk Score	8.28×10^−3^ (1.48)	−4.52×10^−3^ (−1.27)	175
Green view index (GVI)	−1.56×10^−2^ (−1.25)	0.51×10^−2^ (0.64)	
Interaction of GVI and Walk Score	−3.87 ×10^−4^ (−0.49)	2.61×10^−4^ (0.05)	
Percentage of African Americans	2.25 (7.25 ***)	0.32 (1.63)	
Percentage of Asians	−5.60 (−4.00 ***)	−3.25 (−3.71 ***)	
Percentage of Hispanics	0.70 (0.84)	0.88 (1.64)	
Per-capita income	−2.48×10^−5^ (−1.41)	3.08×10^−5^ (2.74 **)	
Percentage of people with a Bachelor’s or higher degree	−4.40 (−4.02 ***)	−3.24 (−4.63 ***)	
Percentage of people without a high-school diploma	2.41 (2.50 *)	0.80 (1.29)	
Moran’s *I*	0.05 (0.12)	−0.01 (0.83)	
F-statistic	64.3	14.7	
Adjusted R-squared	0.77	0.41	

* Association is significant at the 0.05 level (two-tailed); ** association is significant at the 0.01 level (two-tailed); *** association is significant at the 0.001 level (two-tailed).

**Table 3:** The statistical regression analysis result for females and males of the middle-aged group (age 30–50).

Independent Variables	Coefficients		*N*
	Female (*t*-Value)	Male (*t*-Value)	
Walk Score	8.73×10^−3^ (1.72)	−8.94×10^−3^ (−2.52 *)	175
Green view index (GVI)	−3.06×10^−2^ (−2.68 **)	2.48×10^−2^ (3.12 **)	
Interaction of GVI and Walk Score	−9.15×10^−4^ (−1.28)	−4.65×10^−4^ (−0.94)	
Percentage of African Americans	3.38 (11.95 ***)	1.10 (5.58 ***)	
Percentage of Asians	−8.81 (−6.92 ***)	−2.99 (−3.36 ***)	
Percentage of Hispanics	2.16 (2.83 **)	1.21 (2.27 *)	
Per-capita income	−3.63×10^−5^ (−2.27 *)	1.07×10^−5^ (0.96)	
Percentage of people with a Bachelor’s or higher degree	−3.21 (−3.22 **)	−3.05 (−4.39 ***)	
Percentage of people without a high-school diploma	3.11 (3.55 ***)	−0.28 (−0.46)	
Moran’s *I*	0.04 (0.16)	−0.03 (0.80)	
F-statistic	117.7	35.7	
Adjusted R-squared	0.86	0.64	

* Association is significant at the 0.05 level (two-tailed); ** association is significant at the 0.01 level (two-tailed); *** association is significant at the 0.001 level (two-tailed).

**Table 4:** The statistical regression analysis result for females and males of the old-aged group (age 51–65).

Independent Variables	Coefficients		*N*
	Female (*t*-Value)	Male (*t*-Value)	
Walk Score	1.43×10^−3^ (0.23)	−9.48×10^−3^ (−1.97 *)	175
Green view index (GVI)	−2.45×10^−2^ (−1.65)	1.18×10^−2^ (1.10)	
Interaction of GVI and Walk Score	−8.57×10^−4^ (−1.01)	−3.55×10^−4^ (−0.53)	
Percentage of African Americans	2.41 (7.12 ***)	−0.26 (−0.99)	
Percentage of Asians	−6.18 (−4.06 ***)	−3.90 (−3.30 **)	
Percentage of Hispanics	2.24 (2.45 *)	0.92 (1.27)	
Per-capita income	−6.04×10^−5^ (−3.16 **)	−1.33×10^−5^ (−0.88)	
Percentage of people with a Bachelor’s or higher degree	1.22 (1.03)	0.02 (0.02)	
Percentage of people without a high-school diploma	1.72 (1.64)	−1.87 (−2.26 *)	
Moran’s *I*	−0.01 (0.75)	0.05 (0.13)	
F-statistic:	31.9	4.5	
Adjusted R-squared:	0.62	0.15	

* Association is significant at the 0.05 level (two-tailed); ** association is significant at the 0.01 level (two-tailed); *** association is significant at the 0.001 level (two-tailed).

**Table 5:** The statistical regression analysis result for females and males of the retiree group (age 66–84).

Independent Variables	Coefficients		*N*
	Female (*t*-Value)	Male (*t-*Value)	
Walk Score	5.14×10^−3^ (0.78)	−2.83×10^−3^ (−0.53)	175
Green view index (GVI)	−4.51×10^−2^ (−3.04 **)	2.17×10^−2^ (1.81)	
Interaction of GVI and Walk Score	−1.39×10^−3^ (−1.50)	−1.10×10^−3^ (−1.47)	
Percentage of African Americans	1.38 (3.75 ***)	−0.77 (−2.57 *)	
Percentage of Asians	−10.03 (−6.20 ***)	−3.91 (−2.92 **)	
Percentage of Hispanics	0.08 (0.09)	−0.32 (−0.39)	
Per-capita income	−9.21×10^−5^ (−4.23 ***)	−3.43×10^−5^ (−2.04 *)	
Percentage of people with a Bachelor’s or higher degree	2.98 (2.30 *)	−1.49 (−1.42)	
Percentage of people without a high-school diploma	2.16 (1.89)	−2.59 (−2.81 **)	
Moran’s *I*	0.05 (0.09)	−0.03 (0.92)	
F-statistic:	23.76	5.38	
Adjusted R-squared:	0.54	0.18	

* Association is significant at the 0.05 level (two-tailed); ** association is significant at the 0.01 level (two-tailed); *** association is significant at the 0.001 level (two-tailed).
